# Comparing the Impact of DOACs and Warfarin on Below-the-Knee Autologous Vein Bypass Patency in Peripheral Artery Disease: A Retrospective Cohort Study with 2-Year Follow-Up

**DOI:** 10.3390/jpm15070292

**Published:** 2025-07-07

**Authors:** Francisca Frias, Günay Kalender

**Affiliations:** 1 Department of Vascular and Endovascular Surgery, Vivantes Hospital Neukoelln, 12351 Berlin, Germany; guenay.kalender@vivantes.de; 2 Science & Research Department, Brandenburg Medical School Theodor Fontane, 16816 Neuruppin, Germany

**Keywords:** below-the-knee bypass, anticoagulation, PAD

## Abstract

**Background:** Peripheral artery disease (PAD) affects over 200 million individuals globally, leading to significant morbidity and mortality. For patients with complex lesions of the superficial femoral artery requiring revascularization, autologous vein bypass surgery remains a viable treatment option. Postoperative anticoagulation is critical for maintaining graft patency, but the optimal choice between direct oral anticoagulants (DOACs) and warfarin remains uncertain. **Objectives**: This study aims to evaluate the patency of below-the-knee autologous vein bypasses in PAD patients receiving either DOACs or warfarin over a two-year period. **Methods**: A retrospective cohort study was conducted at a tertiary care hospital in Berlin, Germany, including patients who underwent femoropopliteal or femorocrural bypass surgery between 2017 and 2022. Patients were divided into two groups based on postoperative anticoagulation therapy: DOACs or warfarin. The primary outcome was graft patency at 24 months. **Results**: Out of 192 patients, after applying exclusion criteria, 36 were analyzed. The mean bypass patency was longer in the warfarin group (18.3 months) compared to the DOAC group (14.3 months). However, the log-rank test *p*-value (0.524) suggests that this difference is not statistically significant. Given the log-rank test’s limitations in accounting for confounders, a multivariable Cox regression was performed, including age, sex, comorbidities, bypass type and antiplatelet use. The model (Omnibus *p* = 0.93) showed no statistically significant effect for any variable. **Conclusions**: DOACs appear to be a viable alternative to warfarin in maintaining graft patency in below-the-knee autologous vein bypasses for PAD patients. The lack of a need for regular INR monitoring with DOACs may offer a practical advantage in clinical settings.

## 1. Introduction

Peripheral artery disease (PAD) is the preferred term for partial or complete obstruction of one or more peripheral arteries and is estimated to affect more than 200 million people worldwide [1,2]. Although more than 50% of those affected are asymptomatic, PAD can be accompanied by symptoms such as intermittent claudication, rest pain, chronic limb-threatening ischemia (CLTI), and when severe, acute-on-chronic limb ischemia [2,3]. Even in the absence of symptoms, PAD is associated with a notably higher risk of cardiovascular morbidity and mortality, making it a major public health issue [2,4].

CLTI is an end-stage manifestation of systemic atherosclerosis, and it is frequently accompanied by clinically significant cardiovascular disease (CVD), resulting in exceedingly high mortality from stroke and myocardial infraction [5,6].

Antiplatelet agents are strongly recommended for all patients with symptomatic PAD, as they have been shown to reduce the risk of major adverse cardiovascular events (MACEs). Some studies suggest these agents can lower the incidence of such events by approximately 20% [7,8].

Long-term dual antiplatelet therapy (DAPT) or systemic anticoagulation with vitamin K antagonists (VKAs) is not recommended for patients with peripheral artery disease (PAD), and the role of direct oral anticoagulants (DOACs) is still under investigation [9]. The Cardiovascular Outcomes of People Using Anticoagulation Strategies (COMPASS) trial demonstrated that low-dose rivaroxaban (an oral factor Xa inhibitor) combined with aspirin reduced major adverse cardiovascular events (MACEs) and major adverse limb events (MALEs) compared to aspirin alone, though it was associated with a small but statistically significant increase in bleeding risk [10]. However, further studies are needed to clarify these findings.

CLTI is associated with advanced age und multiple comorbidities, and the goal of treatment includes relief of pain, healing of wounds and preservation of functional limbs. However, not all patients are suitable for a revascularization, since it may incur significant morbidity and mortality [11,12]. This is the reason why the estimation of risk and life expectancy plays a critical role in the chosen revascularization strategy.

Although there is no recommended model to use when evaluating the potential of a patient’s revascularization, the stating of the limb is essential and the use of the WIfI classification is recommended [13,14]. All symptomatic patients with severe ischemia (e.g., WIfI grade 3) should be considered for revascularization, assuming that they are suitable candidates for limb salvage [12].

In recent years, the range of minimally invasive techniques for treating complex disease patterns has expanded significantly, prompting some to support an “endovascular-first” approach for most CLTI patients, with bypass surgery considered only as a secondary option. However, existing evidence strongly supports a selective revascularization approach tailored to specific clinical and anatomical scenarios. A systematic review, in fact, indicated comparable mortality and amputation outcomes, with bypass surgery showing even better expected patency rates [15]. Bradbury et al., through the BASIL trial, suggest that patients with severe limb ischemia expected to live at least two years may benefit more from bypass surgery involving veins, while those with shorter life expectancy or no suitable vein are likely better served by balloon angioplasty due to the limited long-term benefit from surgery, higher surgical risks, and lower short-term costs [16].

In a daily setting at a tertiary care hospital, we continue to select a significant number of patients for bypass revascularization and one of the biggest challenges and areas of uncertainty remains how to determine the optimal postoperative antithrombotic regimen. To prevent graft occlusion, patients are usually treated with either an antiplatelet or antithrombotic drug, or a combination of both. Dörffler-Melly et al. concluded that the use of platelet inhibitors led to improved venous and artificial graft patency compared to no treatment [17]. However, an analysis based on graft type revealed that patients with prosthetic grafts were more likely to benefit from platelet inhibitor therapy than those with venous grafts [17]. Geraghty et al. concluded that patients receiving infrainguinal venous grafts are more likely to benefit from treatment with VKAs rather than platelet inhibitors [18]. Although the studies mentioned suggest various approaches, clear guidelines for selecting the most appropriate anticoagulant following bypass surgery remain lacking. Given the heterogeneity of PAD patients, personalized anticoagulation strategies based on individual risk profiles are essential to optimize graft patency while minimizing complications. Although previous studies have shown a comparable bleeding risk between DOACs and VKAs [19], rivaroxaban at higher doses has been associated with a significant increase in bleeding events [20]. This underscores the importance of tailoring anticoagulation choices to each patient’s thrombotic and hemorrhagic risk profile, rather than applying a uniform strategy.

The aim of this study is to evaluate, over a two-year follow-up period, the patency of below-the-knee autologous vein bypass using two different antithrombotic therapies—DOACs and warfarin—and to determine whether there are significant differences between the two treatments.

## 2. Methods

### 2.1. Study Design and Setting

This retrospective cohort study was conducted at Hospital Vivantes Neukoelln, a tertiary care hospital in Berlin, Germany, analyzing data from patients who underwent femoropopliteal or femorocrural bypass surgeries between 2017 and 2022. Autologous vein grafts are considered superior to prosthetic grafts in peripheral arterial bypass surgery, particularly for below-the-knee procedures, due to their higher long-term patency rates [21]. To ensure a more homogeneous and comparable study population, patients who underwent prosthetic or composite bypass surgery were excluded. Data were extracted from electronic medical records and surgical databases, with a follow-up duration of 24 months. The study adhered to the Strengthening the Reporting of Observational Studies in Epidemiology (STROBE) guidelines to ensure methodological rigor and transparency.

### 2.2. Participants

#### 2.2.1. Inclusion Criteria

Patients who underwent below-the-knee bypass surgery (femoropopliteal or femorocrural bypass) for symptomatic PAD presenting with complex superficial femoral artery (SFA) lesions during the study period were included in the cohort. The following inclusion criteria were applied: patients diagnosed with symptomatic PAD (Rutherford class ≥ 3); presence of complex TASC-C and D SFA lesions; having undergone femoropopliteal or femorocrural autologous vein bypass surgery between 2017 and 2022; and postoperative anticoagulation with either DOACs (e.g., rivaroxaban, apixaban, edoxaban, dabigatran) or warfarin.

#### 2.2.2. Exclusion Criteria

Patients were excluded from the study if they received prosthetic grafts such as PTFE, Dacron, Omniflow, or composite bypasses. Additionally, those who underwent popliteopopliteal bypass procedures or experienced graft occlusion within the first week post-surgery were not included, as this was considered a failure due to surgical factors rather than long-term anticoagulation therapy. Patients managed exclusively with antiplatelet therapy without anticoagulation, as well as those lost to follow-up or who died within the first month, were also excluded.

### 2.3. Participant Selection

**Figure 1:**
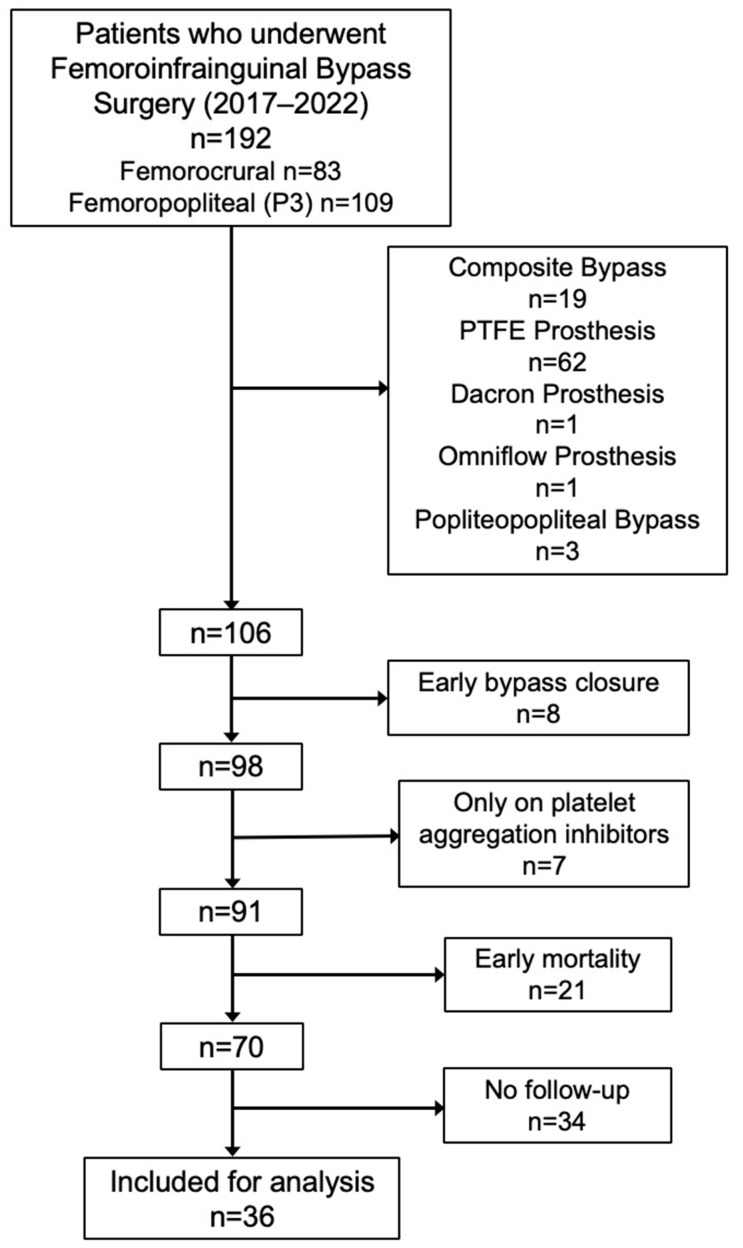
Flow diagram of participant enrollment and exclusion.

A total of 192 patients underwent femoroinfragenual bypass surgery between 2017 and 2022, including 83 femorocrural and 109 femoropopliteal (P3) bypasses. Of these, 83 patients were excluded: 19 had composite bypass implantation, 62 had a prosthetic bypass (PTFE), 1 had a Dacron prosthesis, and 1 had an Omniflow prosthesis. Three patients were excluded due to popliteopoliteal bypass. Seven patients received only antiplatelet therapy without anticoagulation (either DOAC or warfarin). Twenty-one patients died within the first month and were thus excluded. Of these, 12 patients died in the ICU due to respiratory complications (including 3 cases of COVID-19 pneumonia), 3 patients succumbed to multi-organ failure secondary to sepsis, 3 died from hemorrhagic shock, and 1 patient died immediately postoperatively. Two additional patients died after hospital discharge. Finally, 34 patients did not attend the first follow-up appointment, resulting in 36 participants who met all inclusion criteria and completed the study (Figure 1).

### 2.4. Variables

In this retrospective cohort study, we examined both independent and dependent variables to assess the impact of anticoagulant therapy on graft patency following femoropopliteal or femorocrural bypass surgery. The primary independent variable was the type of postoperative anticoagulant therapy administered. Patients received either vitamin K antagonist (warfin) or direct oral anticoagulants (DOACs), such as rivaroxaban, apixaban, edoxaban, or dabigatran. The dependent variable was the graft patency at 24 months, defined as the absence of significant stenosis—characterized by a peak systolic velocity (PSV) greater than 200 cm/s on duplex ultrasonography—or occlusion. To control for potential confounding factors, the following covariates were considered: demographic factors like age and sex; clinical characteristics, like the presence of comorbidities such as diabetes mellitus, hypertension, hypercholesterolemia, renal insufficiency, and atrial fibrillation; surgical details, like the type of bypass procedure performed (femoropopliteal or femorocrural); and postoperative care, like the use of concomitant antiplatelet agents. These variables were meticulously collected from patient medical records to ensure comprehensive analysis and to adjust for potential confounders in the assessment of the relationship between anticoagulant therapy and graft patency. Subgroup analyses were also performed to explore potential differences in treatment response among patient subgroups, supporting a more personalized approach to anticoagulation management.

### 2.5. Data Measurement

The primary outcome of this study was graft patency at 24 months, defined as the absence of significant stenosis or complete occlusion. Graft patency was assessed using duplex ultrasonography. Patients undergoing anticoagulant therapy with DOACs or warfarin were compared. Postoperative follow-up occurred approximately at 3, 6, 12, 18, and 24 months, or at another timepoint if clinical symptoms indicative of bypass closure developed, such as acute pain, reduced pain-free walking distance, or non-healing wounds.

### 2.6. Bias

In this retrospective cohort study, several potential sources of bias were identified that could influence our findings. Selection bias may have been introduced due to the specific inclusion and exclusion criteria, which could limit the generalizability of our results. Information bias is a concern, as reliance on medical records for data collection may have led to inaccuracies or inconsistencies. The relatively small sample size increases the risk of type II errors, potentially affecting the statistical power of our analyses. Additionally, confounding factors, such as variations in patient comorbidities, surgical techniques, and postoperative care, could have influenced the observed outcomes. To mitigate these biases, we employed rigorous data extraction protocols, ensured consistent follow-up procedures, and utilized statistical adjustments, including Cox proportional hazards modeling, to account for potential confounders. Despite these efforts, the inherent limitations of a retrospective design and small sample size necessitate cautious interpretation of the results.

### 2.7. Study Size

Between 2017 and 2022, 192 patients underwent femorocrural (83 patients) or femoropopliteal (109 patients) bypass surgeries. After applying exclusion criteria, 36 patients remained eligible for analysis. This relatively small sample size may affect the statistical power of the study, a limitation that was considered during data interpretation.

### 2.8. Statistical Methods

The primary outcome—graft patency at 24 months—was analyzed using Kaplan–Meier survival curves, with differences between the DOAC and warfarin groups assessed using the log-rank test. To adjust for potential confounding variables, a multivariate Cox proportional hazards model was applied, incorporating diabetes mellitus, hypertension, dyslipidemia, atrial fibrillation, renal insufficiency, smoking status, concomitant antiplatelet therapy, bypass type (femorocrural or femoropopliteal), sex, and age as covariates. Additionally, univariate Cox regression analyses were conducted to explore the individual associations between each of these variables and the occurrence of graft failure. Baseline characteristics between the two anticoagulation groups (DOAC vs. warfarin), as well as between patients with and without the primary endpoint, were compared using appropriate statistical tests. For the continuous variable age, a Shapiro–Wilk test was first used to assess normal distribution, followed by Levene’s test for equality of variances, and then an independent-samples t-test. Categorical variables, including sex, hypertension, antiplatelet use, hypercholesterolemia, renal insufficiency, atrial fibrillation, smoking status, diabetes mellitus, and bypass type were analyzed using Fisher’s Exact Test or chi-square test. Hazard ratios (HRs) with 95% confidence intervals (CIs) were reported for all Cox regression models, and statistical significance was defined as a *p*-value of less than 0.05. All statistical analyses were carried out using IBM SPSS Statistics, version 26.

## 3. Results

### 3.1. Descriptive Data

Among the 36 eligible patients, 20 were in the warfarin group and 16 in the DOAC group (comprising 9 on rivaroxaban, 5 on apixaban, and 2 on dabigatran).

The median age was 67.7 years (SD = 8.05) in the warfarin group and 71.94 years (SD = 8.77) in the NOAC group. Females accounted for 35% of the warfarin group and 18.75% of the DOAC group. In the warfarin group, 45% underwent femorocrural bypass and 55% femoropopliteal bypass; in the DOAC group, 37.5% had femorocrural bypass and 62.5% femoropopliteal bypass. Hypertension was present in 80% of warfarin-treated patients and 100% of DOAC-treated patients, while diabetes mellitus occurred in 25% and 50%, respectively. Atrial fibrillation was absent in all warfarin group patients but present in 56.25% of those in the DOAC group. Active smoking was reported in 70% of the warfarin group and 62.5% of the DOAC group. Dyslipidemia with hypercholesterolemia was present in 25% of the warfarin group and 12.5% of the DOAC group. Although the 2019 guidelines on dyslipidaemias recommend an LDL-C target below 55 mg/dL for patients with peripheral artery disease (PAD) due to their very high cardiovascular risk, in this study, hypercholesterolemia was defined as an LDL-C level greater than 100 mg/dL at the time of surgery. Chronic renal failure—defined as stage 3a or higher (eGFR < 59 mL/min/1.73 m^2^)—was present in 20% of warfarin patients and 37.5% of DOAC patients. Antiplatelet co-administration was observed in most patients—80% in the warfarin group and 93.75% in the DOAC group. Coronary artery disease (CAD) was present in 44.4% of the cohort, which is consistent with reports that up to 60% of PAD patients have coexisting CAD [22] and 12.5% of CAD patients were treated with anticoagulation alone (DOAC or warfarin), without antiplatelet therapy.

After appropriate tests were performed to assess statistical differences in baseline clinical characteristics of both groups (DOAC vs. warfin), no significant difference in mean age was found (*p* = 0.141, Table 1). Categorical variables were analyzed and no statistically significant differences were found between the groups, except for atrial fibrillation, which was significantly more common in the DOAC group (Table 1). This likely reflects current clinical guidelines, which favor DOACs over warfarin for atrial fibrillation due to their improved safety profile and ease of use.

These results suggest that the two groups were largely comparable at baseline, supporting the validity of subsequent analyses regarding graft patency outcomes. Notably, none of the patients in the warfarin group had a mechanical heart valve. Additionally, no patients in the study had a known hypercoagulable state, active malignancy, or other indications for anticoagulation such as deep vein thrombosis or pulmonary embolism.

### 3.2. Outcome Data

In the warfarin group, 6 patients were censored due to loss to follow-up: 3 at 6 months, 2 at 12 months, and 1 at 18 months. In the DOAC group, only one patient was censored during the 24-month period, after the 6-month follow-up consultation. There were a total of 16 events of bypass failure, 8 in the warfarin group and 8 in the DOAC group (Table 1). Of these, 8 patients underwent successful recanalization with percutaneous transluminal angioplasty due to high-grade stenosis of the proximal (4 patients) or distal (4 patients) anastomosis. An additional three patients received catheter-directed thrombolysis, which also resulted in successful recanalization.

### 3.3. Main Results

**Figure 2:**
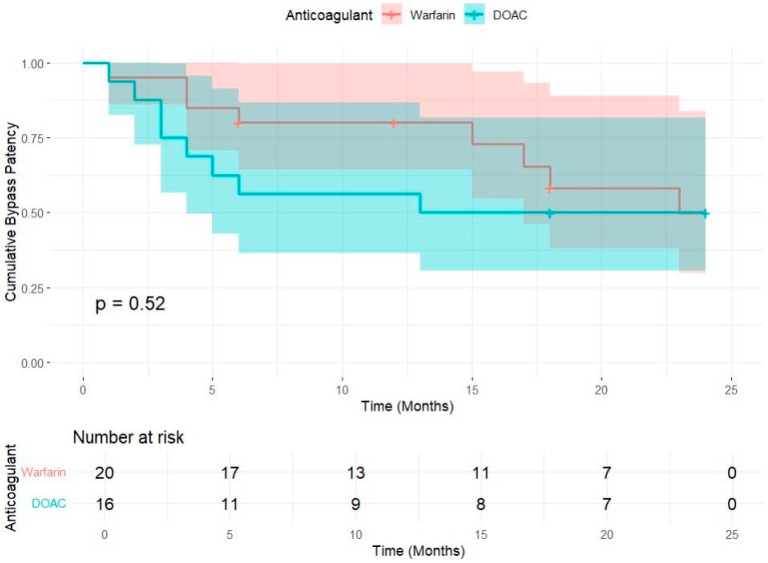
Kaplan–Meier curves for below-the-knee bypass patency by treatment group.

The mean bypass patency was longer in the warfarin group (18,267 months) compared to the DOAC group (14,313 months) (Table 1 and Table 2). However, the log-rank test *p*-value (0.524) suggests that this difference is not statistically significant. The confidence intervals overlap, also indicating a lack of strong evidence for a difference in bypass patency between the groups (Table 1). The survival analysis indicated a trend toward reduced patency in the DOAC group compared to the warfarin group. Specifically, during the first year post-surgery, the DOAC group exhibited a higher incidence of bypass closures or significant stenosis. However, by the 24-month mark, the Kaplan–Meier curves for both groups converged, resulting in similar patency rates at the end of the observation period (Figure 2). Recognizing that the log-rank test (Table 3) is a univariate analysis that does not account for potential confounding factors such as age, comorbidities, or graft type, a multivariable survival analysis was conducted using a Cox proportional hazards regression model. This model included variables such as age, sex, comorbidities (arterial hypertension and diabetes mellitus), type of below-the-knee autologous bypass (femorocrural or femoropopliteal), and the use of concomitant antiplatelet therapy (specifically, 100 mg of aspirin daily). The Omnibus Tests of Model Coefficients yielded a non-significant *p*-value of 0.931 (Table 4), indicating that the model did not significantly predict the occurrence of events. Furthermore, analysis of each variable revealed no statistically significant influence on event occurrence in either group, as shown in the multivariable Cox proportional hazards analysis in Table 5. The co-variables bypass type, antiplatelet co-administration, and current smoking exhibited negative regression coefficients, suggesting that although no statistically significant *p*-values were found, the survival time tended to be longer in the femoropopliteal bypass group, the antiplatelet co-administration group, and the smokers group. In contrast, the survival time was shorter in patients with diabetes, hypertension, atrial fibrillation, and kidney failure. The female group demonstrated a trend toward shorter survival times until the occurrence of the event, as did the DOAC group. Age had a minimal influence on event occurrence, as indicated by a regression coefficient close to 0. To evaluate the individual effect of each variable without accounting for potential confounders, univariate Cox proportional hazards models were also performed (Table 6). These analyses similarly did not reveal any statistically significant associations. Further comparison of baseline characteristics between patients who experienced the primary endpoint and those who did not also revealed no baseline differences, with all *p*-values exceeding 0.05, suggesting a balanced distribution of risk factors across both groups (Table 7).

The plot shows cumulative bypass patency for warfarin (blue) and DOAC (yellow) over a 24-month follow-up. Shaded regions represent 95% confidence intervals, and the risk table below displays the number of patients remaining at risk at each time interval. The log-rank test *p*-value of 0.52 indicates no statistically significant difference in patency between the two groups.

**Table 4:** Omnibus test for Cox proportional hazards model fit.

−2 Log Likelihood	Chi-Square	Dif	Sig
102.014	2.023	7	0.959

Overall statistical test assessing the explanatory power of the Cox proportional hazards regression model. A non-significant *p*-value suggests the included predictors do not collectively account for significant variation in bypass patency outcomes.

**Table 5:** Multivariable Cox proportional hazards analysis of factors possibly affecting bypass patency.

					95% CI for Exp(B)	
	B	df	Sig	Exp(B)	Lower	Upper
Anticoagulation	0.145	1	0.857	1.156	0.238	5.618
Bypass Type	−.343	1	0.651	0.710	0.161	3.131
Sex	0.154	1	0.809	1.167	0.333	4.090
Antiplatelet Coadministration	−0.205	1	0.796	0.814	0.171	3.876
Diabetes Mellitus	0.160	1	0.824	1.173	0.288	4.787
Arterial Hypertension	0.368	1	0.755	1.444	0.144	14.482
Age	0.037	1	0.392	1.038	0.953	1.130
Atrial Fibrillation	0.281	1	0.741	1.324	0.252	6.967
Current Smoking	−0.443	1	0.446	0.642	0.206	2.005
Dyslipidemia	0.792	1	0.257	2.208	0.562	8.681
Kidney Failure	0.019	1	0.889	1.097	0.297	4.055

Regression coefficients (B), hazard ratios (Exp[B]), and 95% confidence intervals were calculated for each variable included in the Cox proportional hazards model. None of the examined factors—type of anticoagulation, bypass type, sex, antiplatelet co-administration, diabetes mellitus, hypertension, age, atrial fibrillation, current smoking, dyslipidemia, or renal insufficiency—demonstrated a statistically significant association with bypass failure. The reference categories used in the model were DOAC administration, femoropopliteal bypass, female sex, presence of antiplatelet co-administration, presence of diabetes mellitus, presence of arterial hypertension, presence of atrial fibrillation, active smoking, presence of dyslipidemia, and presence of kidney failure. Age was included as a continuous variable, expressed in years.

**Table 6:** Univariable Cox proportional hazards analysis of factors possibly affecting bypass patency.

					95% CI for Exp(B)	
	B	df	Sig	Exp(B)	Lower	Upper
Bypass Type	0.065	1	0.898	1.067	0.397	2.869
Sex	−0.167	1	0.773	0.846	0.272	2.632
Antiplatelet Coadministration	−0.303	1	0.636	0.738	0.210	2.594
Diabetes Mellitus	0.057	1	0.912	1.059	0.384	2.917
Arterial Hypertension	0.506	1	0.625	1.658	0.218	12.631
Age	0.037	1	0.253	1.038	0.974	1.107
Atrial Fibrillation	0.493	1	0.362	1.638	0.568	4.725
Current smoking	−0.215	1	0.677	0.806	0.292	2.224
Dyslipidemia	0.561	1	0.334	1.752	0.561	5.469
Kidney failure	0.202	1	0.708	1.224	0.424	3.532

Regression coefficients (B), hazard ratios (Exp[B]), and 95% confidence intervals for each variable in the Cox model. None of the included factors—bypass type, sex, antiplatelet co-administration, diabetes, hypertension, age, atrial fibrillation, current smoking, dyslipidemia, or kidney failure—reached statistical significance in predicting bypass failure. The reference categories used in the model were DOAC administration, femoropopliteal bypass, female sex, presence of antiplatelet co-administration, presence of diabetes mellitus, presence of arterial hypertension, presence of atrial fibrillation, active smoking, presence of dyslipidemia, and presence of kidney failure. Age was included as a continuous variable, expressed in years.

**Table 7:** Statistical analysis testing clinical characteristics of both groups (event of bypass failure vs. event): independent-samples t-test for age; Fisher’s Exact Test for sex, hypertension, antiplatelet co-administration, hypercholesterolemia, renal insufficiency, and atrial fibrillation; chi-square test for current smoking status, diabetes mellitus, and bypass type.

Variable		No Event (n = 20)	Event (n = 16)	*p*-Value
Age		67.95 (SD = 9.02)	71.63 (SD = 7.65)	0.203
Sex	Male	14	12	1.0
	Female	6	4	
Diabetes	No	13	10	0.877
	Yes	7	6	
Hypertension	No	3	1	0.613
	Yes	17	15	
Bypass Type	Femorocrural	10	7	0.709
	Femoropopliteal	10	9	
Antiplatelet Coadministration	No	2	3	0.637
	Yes	18	13	
Atrial Fibrillation	No	16	11	0.470
	Yes	4	5	
Current Smoking	No	6	6	0.635
	Yes	14	10	
Dyslipidemia	No	17	12	0.675
	Yes	3	4	
Kidney Failure	No	15	11	0.722
	Yes	5	5	

No statistically significant differences were found between the two groups for any of the tested baseline variables.

## 4. Discussion

### 4.1. Key Results

Our study compared the two-year patency rates of autologous vein bypasses in patients receiving either warfarin DOACs. The Kaplan–Meier survival analysis revealed that the mean bypass patency was longer in the warfarin group (18.3 months) compared to the DOAC group (14.3 months); however, this difference was not statistically significant (*p* = 0.524). Notably, during the first year post-surgery, the DOAC group experienced a higher incidence of bypass closures or significant stenosis. By the 24-month mark, the patency rates between the two groups converged. Multivariable analysis using a Cox proportional hazards regression model, which accounted for factors such as age, sex, comorbidities, type of bypass, and concomitant antiplatelet therapy, did not identify any variables with a statistically significant impact on event occurrence.

### 4.2. Limitations

Several limitations should be considered when interpreting our findings. The relatively small sample size may limit the statistical power to detect significant differences between groups. Additionally, the observational nature of the study introduces potential biases, such as selection bias and unmeasured confounding variables, which could influence the results. Furthermore, the study’s reliance on routine clinical follow-up may have led to the underreporting of asymptomatic graft failures. Additionally, graft failure (event) was defined as a composite outcome of either complete occlusion or high-grade stenosis, which may introduce variability in the clinical relevance of recorded events. The follow-up period was also limited to 24 months, potentially missing later failures that could impact the interpretation of long-term outcomes.

### 4.3. Interpretation and Generalisability

In routine vascular surgery practice, autologous vein bypass remains a viable option for patients with complex lesions of the superficial femoral artery requiring revascularization. The choice of postoperative anticoagulant therapy is a critical consideration, especially given the lack of specific guidelines on this topic. Although some studies have suggested that warfarin may improve graft patency, a 2022 study by Kim et al. found that, among patients undergoing femoropopliteal bypass grafting, no combination of anticoagulants or antiplatelet medications was associated with better graft patency [23].

Our study contributes to the ongoing discussion regarding postoperative anticoagulation strategies in patients undergoing below-the-knee autologous vein bypass surgery. Although no statistically significant differences were observed between the warfarin and DOAC groups over a 24-month period, the practical advantages of DOACs—such as fixed dosing and the elimination of routine INR monitoring—support their growing use in clinical practice and highlight the importance of individualized care.

Most patients in the warfarin group in this cohort had their anticoagulation therapy initiated nearly a decade ago, reflecting older treatment protocols. In contemporary practice, we observe a trend toward the use of DOACs. This shift is especially relevant for patients with uncertain adherence, as warfarin requires strict INR monitoring (target range 2.0–3.0), and poor compliance may result in either subtherapeutic anticoagulation or excessive bleeding—both of which could negatively affect graft patency.

Renal function remains a key consideration when choosing a DOAC. Although formal guidelines specific to this population are lacking, there is consensus that apixaban 2.5 mg twice daily is appropriate for patients with moderate renal impairment (creatinine clearance between 30 and 50 mL/min). No DOAC is currently recommended for patients with creatinine clearance below 15 mL/min. In contrast, warfarin is initiated at similar doses and managed similarly in patients with kidney impairment as in the general medical population.

As previously noted, a substantial proportion of patients with PAD also have concomitant CAD [22]. While antithrombotic therapy is essential in both groups, the optimal strategy remains under debate and must be carefully balanced against bleeding risk. High bleeding risk (HBR) includes factors such as prior stroke, recent gastrointestinal bleeding, advanced age, or renal impairment [24]. This is consistent with findings from the ARRIVE trial [25], which demonstrated only marginal net benefit of aspirin in primary prevention, accompanied by an increased risk of major bleeding. These findings highlight the importance of individualized treatment decisions. In this context, the co-administration of antiplatelet therapy should also be personalized based on the patient’s overall risk profile.

Our suggested clinical approach, supported by the findings of this study, is to initiate DOAC therapy—typically rivaroxaban 20 mg once daily—in patients without significant renal impairment. For patients already receiving anticoagulation for atrial fibrillation, continuation of their current regimen is generally appropriate. In elderly patients with renal impairment and an elevated bleeding risk (e.g., due to frequent falls), reduced-dose apixaban may be considered. In compliant patients with renal impairment but low bleeding risk, or in those with mechanical heart valves, warfarin remains a viable and effective option. The co-administration of antiplatelet therapy is similarly nuanced. While the COMPASS trial recommends combining aspirin with low-dose rivaroxaban [11], we individualize this decision in clinical practice based on bleeding risk. In patients with a high bleeding risk, monotherapy with an anticoagulant is often preferred—an approach that was applied to five patients in this cohort. However, this remains an area of clinical uncertainty, with no established consensus or guidelines currently available.

A personalized treatment approach—taking into account adherence, comorbidities, renal function, bleeding risk, and other indications for anticoagulation—is essential for optimizing outcomes in this complex population.

In this cohort, DOACs demonstrated comparable effectiveness to warfarin in preventing graft stenosis or occlusion. This findings may extend to similar patient populations undergoing autologous vein bypass for complex superficial femoral artery lesions. However, the limited sample size, observational design, and potential for bias highlight the need for caution when generalizing these results to broader clinical practice. The higher incidence of events in the DOAC group during the first year warrants further investigation to determine if this trend is clinically significant. Larger, prospective, randomized trials are needed to determine whether DOACs or warfarin provide superior long-term graft patency in patients undergoing infrainguinal bypass surgery.

## Figures and Tables

**Table 1:** Statistical analysis testing clinical characteristics of both groups (warfin vs. DOAC): independent-samples *t*-test for age (in years); Fisher’s Exact Test for sex, hypertension, antiplatelet co-administration, hypercholesterolemia, renal insufficiency, and atrial fibrillation; chi-square test for current smoking status, diabetes mellitus, and bypass type. * indicates that there is a statistically significant value.

Variable		Warfin (n = 20)	DOAC (n = 16)	*p*-Value
Age		67.7 (SD = 8.05)	71.94 (SD = 8.77)	0.141
Sex	Male	13	13	0.456
	Female	7	3	
Diabetes	No	15	8	0.121
	Yes	5	8	
Hypertension	No	4	0	0.113
	Yes	16	16	
Bypass Type	Femorocrural	11	6	0.296
	Femoropopliteal	9	10	
Antiplatelet Coadministration	No	4	1	0.355
	Yes	16	15	
Atrial Fibrillation	No	20	7	0.000 *
	Yes	0	9	
Current Smoking	No	6	6	0.635
	Yes	14	10	
Dyslipidemia	No	15	14	0.426
	Yes	5	2	
Kidney Failure	No	16	10	0.285
	Yes	4	6	

No statistically significant differences were found between the two groups (NOAC and warfarin) for any of the tested baseline variables, except for atrial fibrillation.

**Table 2:** Estimates of mean bypass patency duration.

			95% Confidence Interval	
Anticoagulation	Estimate	Std. Error	Lower Bound	Upper Bound
Warfin	18.267	1.815	14.710	21.824
DOAC	14.313	2.500	9.413	19.212
Overall	16.564	1.540	13.545	19.584

Mean bypass patency times (in months) for warfarin and DOAC groups, including standard errors and 95% confidence intervals (CIs), derived from Kaplan–Meier survival analysis.

**Table 3:** Log-rank test for comparison of bypass patency between warfarin and DOAC groups.

	Chi-Square	Dif	Sig
Log Rank (Mantel–Cox)	0.406	1	0.524

The Log-Rank (Mantel-Cox) test showed no statistically significant difference in bypass patency between the warfarin and DOAC groups over the 24-month follow-up period (χ^2^ = 0.406, df = 1, *p* = 0.524).

## Data Availability

The data used in this study are not publicly available due to ethical restrictions and the need to protect patient confidentiality.

## Abbreviations

The following abbreviations are used in this manuscript:

PAD	Peripheral artery disease
CLTI	Chronic limb-threatening ischemia
VKAs	Vitamin K antagonists
CVD	Cardiovascular disease
MACEs	Major adverse cardiovascular events
DAPT	Dual antiplatelet therapy
MALEs	Major adverse limb events
SFA	Superficial femoral artery
DOACs	Direct oral anticoagulants
CAD	Coronary artery disease
HBR	High bleeding risk
SD	Standard deviation
CIs	Confidence intervals
